# Automated Transverse Crack Mapping System with Optical Sensors and Big Data Analytics

**DOI:** 10.3390/s20071838

**Published:** 2020-03-26

**Authors:** Kwanghee Won, Chungwook Sim

**Affiliations:** 1 Electrical Engineering and Computer Science, South Dakota State University, Brookings, SD 57007, USA; kwanghee.won@sdstate.edu; 2 Department of Civil and Environmental Engineering, University of Nebraska-Lincoln, Omaha, NE 68182, USA

**Keywords:** optical sensor, computer vision, big data pipeline, automated transverse crack mapping, bridge deck

## Abstract

Transverse cracks on bridge decks provide the path for chloride penetration and are the major reason for deck deterioration. For such reasons, collecting information related to the crack widths and spacing of transverse cracks are important. In this study, we focused on developing a data pipeline for automated crack detection using non-contact optical sensors. We developed a data acquisition system that is able to acquire data in a fast and simple way without obstructing traffic. Understanding that GPS is not always available and odometer sensor data can only provide relative positions along the direction of traffic, we focused on providing an alternative localization strategy only using optical sensors. In addition, to improve existing crack detection methods which mostly rely on the low-intensity and localized line-segment characteristics of cracks, we considered the direction and shape of the cracks to make our machine learning approach smarter. The proposed system may serve as a useful inspection tool for big data analytics because the system is easy to deploy and provides multiple properties of cracks. Progression of crack deterioration, if any, both in spatial and temporal scale, can be checked and compared if the system is deployed multiple times.

## 1. Introduction

### 1.1. Motivation

Bridge decks which are directly exposed to traffic loading and chlorides (in cold regions or marine environments) would typically have a shorter service life than other bridge components. Bridge deterioration from bridge decks is the starting point of degradation which can be followed by other structural members (superstructure, supports, and substructure). For such reasons, health monitoring of bridge decks plays an important role in maintaining and increasing the service life of bridges. Bridge decks will typically contain a transverse restrained shrinkage crack which is full-depth across the cross-section. These transverse cracks provide the path for chloride penetration and is the major reason for deck deterioration. For such reasons, collecting information related to crack widths and the spacing of these transverse cracks are important. 

In Nebraska, there are more than 15,000 bridges which the state department of transportation needs to monitor and this is only including bridges that are longer than 20 ft. The Federal Highway Association (FHWA) requires every state agency to update the condition of bridge superstructures and substructures every 1–2 years, and this information is archived as the National Bridge Inventory (NBI) data. Other than the load ratings, most of the records collected from a bi-annual bridge inspection is an output of manual visual inspections. Realizing that the number of bridges that require inspection is more than several thousand, there have been many efforts [1,2,3,4] to develop automated visual inspection systems. The data collected through an automated visual inspection may be less subjective compared to those provided by human inspections. Another helpful aspect of automated inspection is that one can collect the outcomes over a long-term and analyze them in data-intensive ways. This may help predict the progression of deterioration both in temporal and spatial scales. The collected data can also be used in observing the correlation over various inspection records from different bridges that could possibly provide useful information identifying parameters that affect the service life of bridge decks.

### 1.2. Related Work

A typical data processing pipeline for automated crack detection with non-contact optical sensors consists of three major tasks: (1) data acquisition, (2) localization, and (3) crack detection. The possible types of input data for data acquisition are image sequences, GPS, odometer, and LiDAR data. First of all, the data acquisition speed for such systems is dependent on the speed of optical sensors and the moving platform. The ideal system should be able to acquire data in a fast and simple way without obstructing traffic. Secondly, the localization task may typically rely on the GPS and odometer sensor data. Odometer sensor data can provide relative positions along the direction of traffic but not in the transverse direction. It is also well known that GPS signals are not always available (for example, in urban environments) or GPS does not provide the pose information. For such reasons, this study focused on providing an alternative localization strategy in order to re-identify the cracks between periodic inspections. Finally, for the detection task, various approaches have been proposed in previous studies, including a simple image processing or machine learning based approach [1,2,3,4]. Most of the existing crack detection methods are based on low-intensity and localized line-segment characteristics of cracks. This method will not be efficient in successfully categorizing “cracks” and “non-cracks”, specifically if the bridge deck has a tined finish. To improve existing approaches, other characteristics, such as the direction or shape of the cracks, should be considered in a smarter machine learning approach. More recently Deep Learning-based algorithms using convolutional neural networks have been introduced in this domain, which demonstrated promising results [5,6,7,8]. However, the examples of demonstration and implementation of these methods regarding the entire structure in large scale (not for a local detection problem) have been limited. 

The detection and localization tasks will provide crack maps which indicate the location and the size of the cracks. Crack maps generated by human drawings may have errors and they may not necessarily contain all crack width information. In addition, there have been recent studies focusing on crack detection techniques through image processing, and a detailed literature review is provided in one of these studies [9]. However, most of these studies focused on improving crack detection and not necessarily applying their methods for the entire deck, and only providing information for a local region. It would be much more useful to be able to identify where the specific crack is located within the bridge, what the average and maximum crack widths are, how long the cracks are, and what the shape (direction) of the crack is to assess the damage level and causes of them. For example, by observing the shape and directionality, it will be possible to identify whether it is a material or a structural crack. By having the length information, one will be able to identify whether the crack is a primary crack or a secondary crack. Crack widths and spacings would be useful information that inspectors would like to know. Many of the existing crack detection systems are not providing these essential data, which is one of the reasons bridge owners are not fully utilizing these new techniques.

### 1.3. Knowledge Gap

Data Acquisition and Localization

1. The data acquisition speed for such systems is dependent on the speed of optical sensors and the moving platform. The ideal system should be able to acquire data in a fast and simple way without obstructing traffic. Most of the previous studies use either GPS, odometer sensor data or LiDAR data for localization tasks. However, there are locations where GPS signals are not always available for example, in an urban environment. GPS also does not provide pose information. In addition, odometer sensor data can provide relative positions along the direction of traffic but not in the transverse direction perpendicular to the direction of traffic. If one would like to generate a stitched (global) map of an entire bridge deck in an urban area, an alternative localization strategy may be required.

Crack Detection and Measurements

2. Many studies focus on developing crack detection algorithms and demonstrate their methods only for a local region. Implementing these algorithms to a larger scale and for the entire structural member is challenging and rarely demonstrated in these research studies.
3. Most of the crack detection algorithms are relying only on line segments and their intensity. This will limit the information to whether there is a crack or not and not necessarily capturing the shape of a long crack shown in the entire member that a structural engineer would be interested in.
4. Previous research focuses on detecting cracks and not necessarily on making measurements of crack widths and spacings to create a database for future maintenance.

### 1.4. Research Significance

This study proposed a new type of automated crack mapping system that addresses the problems listed in the knowledge gap. The contribution of this research study is as follows:

1. Proposed an alternative localization strategy using multi-view image sequences and used Bag-of-Words (BoW) representation of images to complete the localization without GPS and odometer data.
2. Created a hierarchy of crack pixels and crack segments to detect cracks, and used a circular histogram to capture the orientation of cracks which is not relying on finding line segments.
3. Demonstrated that this algorithm can be implemented for an entire bridge deck member rather than a local region of interest.
4. Created ontologies and schemas for generating databases and providing crack measurement data that can be used for future maintenance and decision making additional to the crack detection.
5. Demonstrated that the big data image pipeline for conducting crack investigations can be used as a reference pipeline/framework for other applications where large amounts of images are used for data collection, data analysis (detection of deficiencies; in this study, cracks), and decision making based on the database provided.

## 2. Proposed Methodology

In this research, we have developed a computer vision-based crack mapping system that can provide all of the essential and critical information (crack location, number of cracks, crack spacings, crack widths, crack length, and shape of the crack) bridge owners would like to archive. The system collects thousands of images from a bridge deck with machine vision cameras attached to a road vehicle that travels in normal traffic speed without obstructing existing traffic. It produces not only a global crack map which provides the location of the cracks, but also the local information for each crack including the orientation, width, and length. 

**Figure 1:**
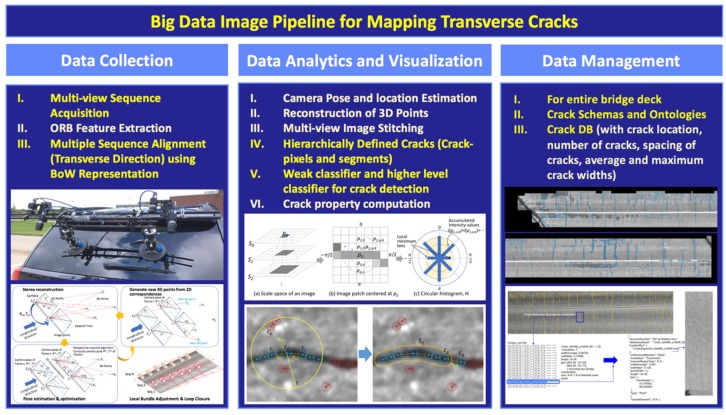
Overall process of the image-based data processing pipeline.

To achieve the resolution of the widths of typical cracks observed in bridge decks (average crack widths measured in bridge decks are approximately 20 mils; 0.020 in.) and to be able to provide the location of each crack, we have employed a multi-view, multi-resolution camera system. The system uses a 3D computer vision-based localization and mapping technique. Two cameras have a wide field-of-view (angle) for localizing frames in both longitudinal and transverse directions. The other two cameras have deeper focal lengths and can capture high resolution (detailed) images of the cracks. The minimum crack width this system can capture is 10 mils (0.010 in.; hairline cracks; half of average crack widths observed in bridge decks). Figure 1 shows the overall process of our big data image processing pipeline that this research proposed for creating transverse crack maps. The big data pipeline is composed of three layers of research activities: (1) data collection (sensing through high-speed multi-view sequence acquisition), (2) data analytics and visualization (crack detection), and (3) data management (ontologies and schemas to create a transverse crack database). Each layer of work may look similar to those of other conventional approaches, but the difference (highlighted in yellow in Figure 1) originates that our system (1) collects multi-view image sequences as input data and uses Bag-of-Words representation of images to complete localization rather than using GPS or odometer data, (2) detects cracks through crack hierarchy and circular histograms rather than through line segments and intensities, and (3) creates a transverse crack database, based on ontologies and schemas that we developed and targets the entire bridge deck. The system is designed to achieve faster deployment and higher accuracy in measurements and mapping cracks in a larger scale. 

One of the challenges in making detailed measurements and localization during high-speed acquisition is that the relative pose (in 3D) between the four cameras and the surface of a bridge deck continuously changes. Therefore, localization is completed by recovering the camera pose and location in 3D for every single frame and reconstructing the 3D surface of the bridge deck, without using GPS data. 

For crack detection, unlike other approaches which are based on a relatively simple definition or assumptions for cracks in images (e.g., line segments with low-intensity pixels), we used multiple hierarchical characteristics to define cracks. At the lower level, we defined a crack pixel that has low-intensity values and has a dominant orientation. At the higher level, we defined a crack segment which is a sequence of crack pixels, or a sequence of crack segments, recursively. According to the hierarchy, we have applied two levels of classifiers. The crack pixels are over-detected by a weak classifier. Once they form crack segments, the higher-level classifier determines the labels based on the features obtained from each crack segment.

Focusing on the needs the bridge owner would be interested in, we created schemas that can be used as a protocol to create crack databases as the final step of our research. The schemas are available at https://github.com/BridgingBigData/bridgehealthschema [10]. 

## 3. Experimental Setup

### 3.1. Multi-View Sequence Acquisition

**Figure 2:**
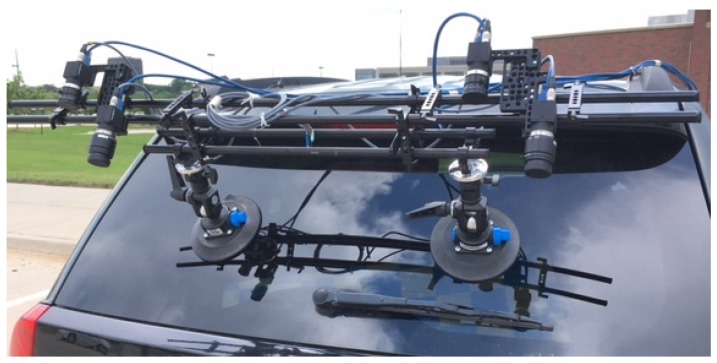
Multi-view camera system mounted on a road vehicle.

We used multiple optical sensors attached to a road vehicle for data acquisition, as shown in Figure 2. Two types of optical sensors (cameras) were used, where one served for localization and the other for crack detection purposes. For image-based localization, two large field-of-view cameras were used to cover a wide surface area (the entire bridge deck) and to compensate the relative pose changes through performing stereo reconstruction of 3D points on the bridge deck in real-scale. The other two cameras were used for crack detection and have a longer focal length to capture the surface of a bridge deck with high resolution images (provides two-times finer resolution than the other pair of cameras).

**Figure 3:**
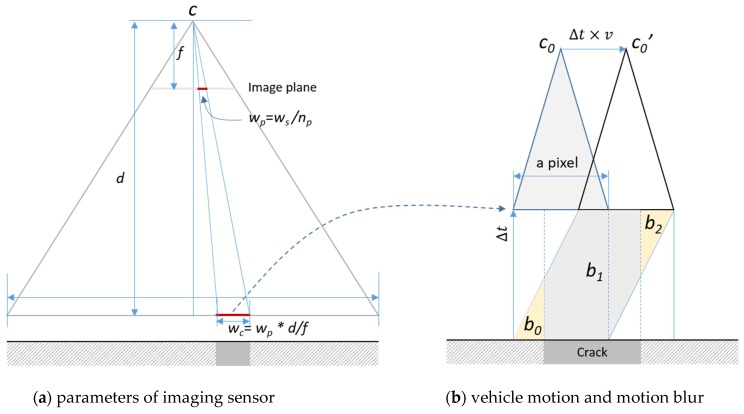
Parameters that control the accuracy of crack measurement.

The typical width of a crack pixel projected on the ground, *w_c_*, should be smaller than the (desired) minimum measurable width of cracks to increase the accuracy of crack measurements, as shown in Figure 3a, where *w_s_* is width of the sensor, *n_p_* is the number of pixels, *f* is the focal length, and *d* is the distance between the focal point and the target surface. Another important factor that affects the accuracy of the measurement is motion blur, caused when the vehicle is moving at high speed. In Figure 3b, *Δt* represents the exposure time and *v* is the speed of the vehicle. During *Δ*t, the proportion (*b_0_*+*b_2_*)/(*Δt×w_c_*) of the value of one pixel is obtained and accumulated from the adjacent area, and this creates blur around the boundary of a crack pixel. The smaller *Δ*t is, the blur will decrease. However, noise will increase under low illumination conditions and the results depend on the changes in ambient lighting. If needed, an additional light source is required to decrease this noise level. 

The four imaging sensors capture multi-view sequences along the direction of traffic. The sensors are synchronized by a trigger signal. The *i*th sequence *s_i_* consists of multi-view frames *f_i,j_*, (0 ≤ *j* < *n_i_*). Each frame has four images *I*_0_, *I*_1_, *I*_0_*^h^*, and *I*_1_*^h^*. The images are undistorted using the distortion parameters. The frames have an order in their sequences, but they do not have information about adjacency to other sequences in transverse direction of the bridge.

We extracted image features for each frame of all sequences before the localization step. The main purpose of extracting features is to find pixel-level correspondences between frames and image-level correspondences among sequences. We have selected ORB (**O**riented FAST [11] and **R**otated **B**RIEF—Binary Robust Independent Elementary Features [12]) which detects FAST (Features from Accelerated Segment Test) corners and produces a binary descriptor of 256 bits for each feature point [13]. It is fast and simple but invariant to orientation changes. The features and descriptors are extracted after warping each image using a homography matrix, which represents a mapping from the image to the ground plane and is obtained by sparse stereo matching [14]. This process decreases the scale differences within a frame or among frames generated by perspective projection or by the motion of the vehicle. This will adjust and minimize the error that can accumulate based on vehicle dynamics. 

### 3.2. Image-Based Localization and Mapping

We performed two levels of image-based localization. First, we aligned the sequences in transverse direction and defined an adjacency graph, *G = (V, E)*, including frames of all sequences. The vertex, *V*, is a set of all frames and a set of all edges, *E* which, consists of edges within a longitudinal sequence, *E_L_*, and edges across transverse sequences, *E_T_* can be expressed as Equation (1). *E_T_* can be obtained by comparing features of a frame to those of all frames of other sequences.

(1) $$E=E_{L}\cup E_{T}{}_{,}E_{L}=\left\{f_{\left(i,j\right)}f_{\left(k,l\right)}|\left|i-k\right|=1,j=l\right\}$$

The time complexity is *O(n^2^m^2^)* where *n* is the number of total frames and *m* is an average number of features in a frame.

**Figure 4:**
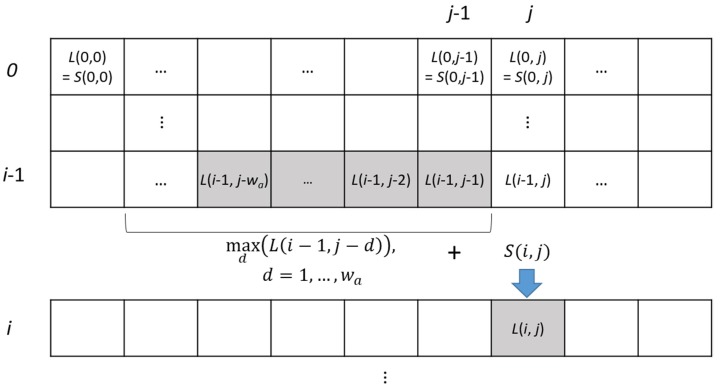
Aggregation of BoW-based similarity score for sequence matching.

Instead of using the brute-force approach, we used image to image comparison with the Bag-of-Words (BoW) representation of images. This method represents each image using a vector which records the presence of certain features within an image as words in a dictionary [15]. By comparing these vectors, we can get a similarity score between the two different images. In addition, we can assume that the two sequences should be matched monotonically in an increasing or decreasing (if sequences are matched in a reverse direction) order, if two sequences are adjacent to each other. We computed the similarity scores within a specified size of window and found the best matches by aggregating the scores through the dynamic programming strategy. The computation was completed for every *k^th^* frame (these frames are defined as key frames), *f_i_’*, *i′* = *ki* of a source sequence to the corresponding *w* frames *f_j_*, j = (*ki* − *w*/2, …, *ki* + *w*/2) of the target sequence, as shown in Figure 4. The aggregated score is defined as follows:

(2) $$L(i,j)=S(i,j)+\underset{d}{\max}\left(L\left(i-1,j-d\right)\right),d=1,\ldots ,w_{a}$$

where, *S(I, j)* contains BoW-based similarity score, *w_a_* is the size of the aggregation window (Figure 4).

The best score between the two sequences was defined as:

(3) $$s=L\left(n_{k},j_{best}\right),where,j_{best}=\underset{j}{\arg\max}\left\{L\left(n_{k},j\right)\right\}$$

where, *n_k_* is the index of the last key frame of the source sequence. The matches can be backtracked from the last match (*n*, *j_best_*). 

**Figure 5:**
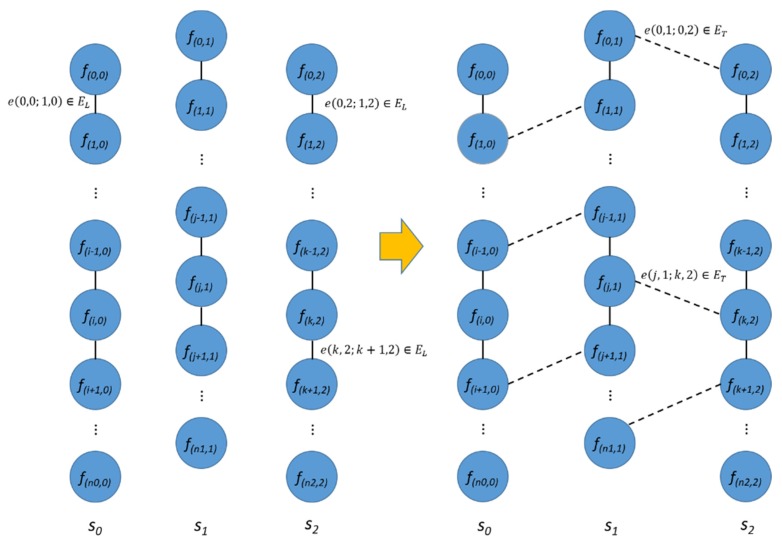
Image sequences and an adjacency graph with sets of edges EL and ET.

For each sequence, the sequences of the *k*-highest scores were considered as the adjacent sequences, and the corresponding matches were included in the set of transverse edges, *E_T_*. These image-level correspondences were further verified on the next step of the localization process. The transverse edges were removed if there was not a sufficient number of matched features, or if the correspondences did not meet the geometric requirements (e.g., a plane homography matrix). Figure 5 shows the sequences and adjacency graph *G* with longitudinal and transverse edges *E_L_* and *E_T_*.

The second step for image-based localization and mapping was conducted to obtain the 3D translation and rotation of each frame, by traversing each node of the adjacency graph, *G* in breath-first order.

**Figure 6:**
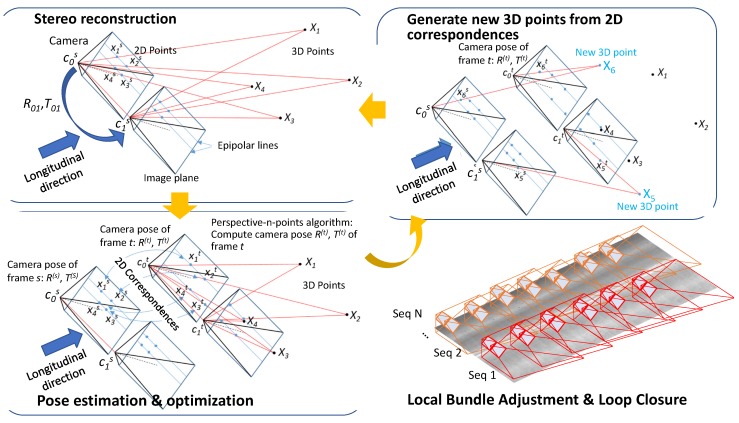
Image-Based localization and mapping.

As illustrated in Figure 6, we reconstructed 3D points using 2D correspondences among views within a frame since our multi-view camera sensors were calibrated. After, a frame has 3D information for enough number of 2D feature points, we can compute the rotation and translation of an adjacent frame by searching for the 2D feature correspondences and solving a perspective-n-point problem.

The projection of a 3D point is represented as the following equation [14]:

(4) $$s\left[\begin{array}{c} x\\ y\\ 1 \end{array}\right]=K\left[R|T\right]X_{i}=\left[\begin{array}{ccc} f_{1} & 0 & c_{x}\\ 0 & f_{2} & c_{y}\\ 0 & 0 & 1 \end{array}\right]\left[\begin{array}{ccc} r_{11} & r_{12} & r_{13}\\ r_{21} & r_{22} & r_{23}\\ r_{31} & r_{32} & r_{33} \end{array}\right]\left[\begin{array}{c} t_{1}\\ t_{2}\\ t_{3} \end{array}\right]\left[\begin{array}{c} X\\ Y\\ Z\\ 1 \end{array}\right]$$

where, *K* is the intrinsic parameter and obtained by calibration, *(x, y)* and *X* are the 2D feature point and the corresponding 3D point. *R* and *T* are the rotation matrix and translation vector, respectively. We computed the *R* and *T* using the ePnP algorithm [14,16] with 2D to 3D correspondences. Then, we optimized both *R* and *T*, by minimizing the following objective function, which is defined using the stereo re-projection error.

(5) $$\underset{R,T}{\min}\sum_{i}D\left(K\left[R|T\right]X_{i}{}_{,}x_{i}^{0}\right)+D\left(K\left[R\left[R_{01}|T_{01}\right]+T\right]X_{i},x_{i}^{1}\right)$$

where, *D*(·,·) is the distance function defined between the projected point and the observed point. *R*_01_ and *T*_01_ is the rotation and translation from the first to the second cameras. *X_i_* is the 3D point and *x_i_*^0^ and *x_i_*^1^ are the observed points in the first and second camera, respectively.

We generated more 3D points using the direct linear triangulation method with 2D correspondences between these two frames that have external camera parameters. This frame by frame estimation may have errors and the reconstructed 3D point should be consistent (by having the similar reprojection errors) for all frames which can observe those points. So, the iteration process requires a bundle adjustment for each key frame to minimize the errors in the estimation of camera poses and 3D points at the same time. The objective function is as follows:

(6) $$g\left(R,T,X\right)=\sum_{i=1}^{n}\sum_{j=1}^{m}v_{ij}D\left(x_{ij,}P\left(K_{i}\left(R_{i},T_{i}\right),X_{j}\right)\right)$$

where, *R* and *T* are the rotations and translations of target frames, *D*(·,·) is the distance function, *P*(*K_i_*(*R_i_*,*T_i_*),*X_j_*) projects a 3D point *X_j_* using a projection matrix defined by the intrinsic matrix *K_i_*, and rotation and translation, *R_i_* and *T_i_*. A visibility function, *v_ij_* represents the visibility of *jth* 3D point, *X_j_*, to the *i*th camera defined by *K_i_*, *R_i_*, and *T_i_*. The set of participating frames, *F_b_*, are selected by building a breadth-first search tree and taking the nodes of the first *l*-levels starting from the root node. The set of 3D points, *P_b_*, consists of all observable 3D points from any frames in *F_b_*. A set of frames, *F_f_*, which can observe any 3D points in *P_b_*, but not in *F_b_*, will also participate in the process, but their values will not be changed (fixed) and they will only contribute to the evaluation of the objective function. In Equation (6), *n* is |*F_b_* U *F_f_*| and *m* is |*P_b_*|, where |·| represents a cardinality of a set.

Because the key frames have both longitudinal and transverse edges in the adjacency graph, there are cycles in the graph. So, the breath-first traversal may visit the same node multiple times if there is a loop. In this case, the pose difference (error) between the estimated pose of the frame before revisiting and the estimated pose along the loop needs to be corrected to minimize the accumulated error. This accumulated loop closing errors can be distributed by the pose graph optimization [17,18]. This iterative process localizes each frame as well as generates the 3D points of the bridge deck. 

### 3.3. Crack Detection

**Figure 7:**
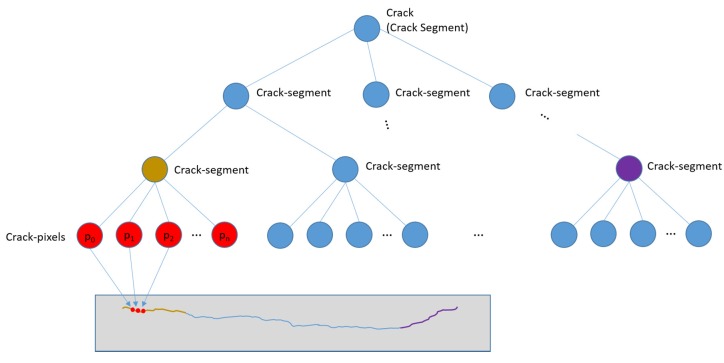
Hierarchy of crack pixels and crack segments.

In order to detect cracks from images, we have defined a crack hierarchy using crack pixels and crack segments, as shown in Figure 7. The crack pixel is the basic unit of the crack within an image. A crack pixel has low-intensity values and has a dominant orientation. A crack segment is an ordered set of crack pixels or it is recursively defined by a set of crack segments. Each crack pixel of a crack segment should have similar orientation with those of the neighboring crack pixels. The relative position to the neighboring crack pixels should be consistent with the orientations of them as well.

(7) c(u,v)=(p(u,v),s,I(p),θ,g(p),w)

where, *p(u, v)* is the position of the crack pixel in the coordinates of the stitched image, *u* and *v* indicate the position in transverse and longitudinal direction, respectively. *s* is the scale of the crack pixel. *I(p)* is the intensity value of the pixel, *θ* is the orientation, *g(p)* is the gradient of pixel values in the perpendicular direction of *θ*, and *w* is the width of the crack. 

Many of the previous research studies assumed that a crack has low intensity and the local shape can be approximated by a line segment. Our definition of a crack element is stricter considering the dominating characteristics of the pixels (low-intensity and a dominant local orientation), but more flexible (less strict) for the shape of the crack. By the definitions of a crack pixel and a crack segment, each crack is represented by a tree structure, as shown in Figure 7. The nodes of the tree are crack segments and the leaf nodes have crack pixels, as shown in the Figure 7. 

**Figure 8:**
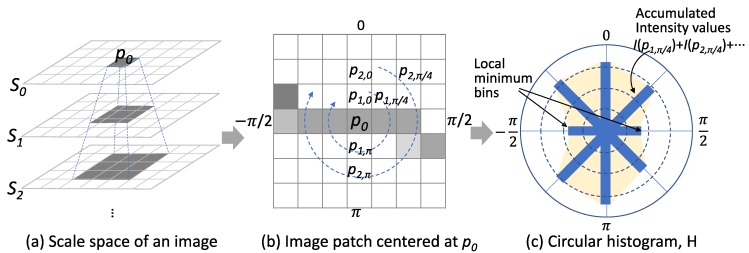
Detection of a crack pixel using an orientation histogram.

The detection of cracks is completed in two steps. In the first step, we detected crack pixels by examining the local image patch centered at each pixel. We prepared a scale-space to take into account different scales of cracks to detect the cracks greater than a single pixel. Then, for each pixel *p_0_*, we built a circular histogram from the image patches of all scales to check whether the pixel has a dominant orientation of low-intensity pixels or not. Each bin of the histogram represents the relative orientation of pixels within the patch. Each histogram accumulates pixel values along the relative direction of the pixels in the patch as illustrated in Figure 8. In the figure, *I*(*p*_1_, _π/4_) represents a pixel value of the pixel that is located at the direction of π/4 and has distance of 1 from the pixel *p_0_*. We determined a dominant orientation by detecting two bins that have local minimums and averaging the corresponding orientations, as shown in Figure 8. Once the orientation was determined, the gradient of pixel values to the perpendicular direction of the orientation was computed. In the figure, the average of |*I*(*p*_0_) − *I*(*p*_1,0_)| and |*I*(*p*_0_) − *I*(*p_1,π_*)| is the value of *g*(*p_0_*). The scale selection was done by comparing *g*(*p*_0_) values of the crack pixels of all scales. The crack pixel that had the maximum *g*(*p*_0_) and the corresponding scale was selected as the value of *s* for the location *p*_0_. This weak classifier inherently produced false-positives because the properties we used for crack pixel detection were not sufficient for describing a crack element.

In the second step, we formed crack segments and classified them into cracks and non-cracks. For each detected crack pixel, we counted the number of supporting crack pixels that satisfies the following conditions: (1) the supporting crack pixels should be within a local window defined by a radius *r*, (2) they should have the similar orientation with that of the crack pixel, (3) the relative direction of the crack pixels with respect to the crack pixel should be consistent with the orientations of themselves and the crack pixel. These three conditions are represented in the following equations:

(8) $$d=\left|p\left(u,v\right)-p\left(u_{i},v_{i}\right)\right|<r$$

(9) $$\left|v_{i}\cdot v_{o}\right|>\cos\left(\theta_{s}\right)$$

(10) $$\min\left(\left|\theta -\theta_{i}\right|,\frac{\pi }{2}-\left|\theta -\theta_{i}\right|\right)<\theta_{s}$$

where, $v_{i}=\left(p\left(u_{i},v_{i}\right)-p\left(u,v\right)\right)/d$, $v_{0}=\left(\cos\left(\theta \right),\sin\left(\theta \right)\right)$, θ is the orientation of the crack pixel *p(u,v)* and *θ_i_* is the orientation of the *i*-th crack pixel *p(u_i_,v_i_)*. *θ_s_* represents the maximum angle difference.

**Figure 9:**
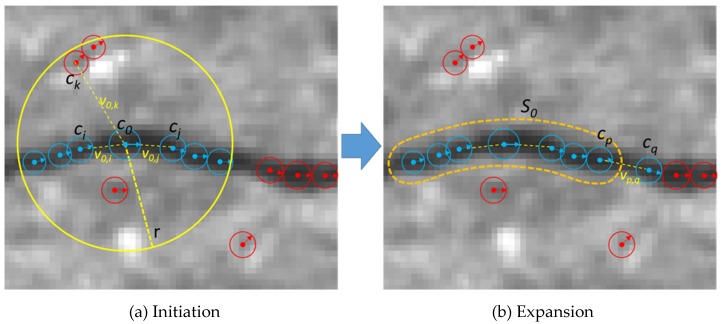
Crack segment generation.

We initialized a crack segment using a crack pixel that has the maximum number of supporting pixels within a local window. This process is illustrated in Figure 9. For a crack pixel *c*_0_, the pixels *c_i_* and *c_j_* (blue-colored pixels) are the supporting pixels, but *c*_k_ is not a supporting pixel of *c*_0_ because the vector *v_0,k_* and the orientation of *c*_0_ are different. Crack segments will have a sorted list of crack pixels, location, average orientation, length, and an average width. A sorted list of crack pixels is obtained by projecting the pixels onto the principle axis of the crack pixels (the direction that the crack pixels are aligned with). We expanded the initial segments in two directions by adding crack pixels that were not assigned to any crack segments. Two end-points are the first and last elements of the sorted list of crack pixels. In Figure 9, *c_p_* is one of the end-points of an initial crack segment *s*_0_, and *c_q_* is a candidate crack pixel which can be included into *s_0_*. The crack pixels that are not assigned to any crack segments will be discarded. In the next step, crack segments are compared and linked if end-points of two crack segments are close enough and the average orientations of two crack segments are similar (when it is less than *θ_s_*). The existing approaches also connect locally detected cracks which are mostly consisted of line segments, but the crack segment defined in our study is an ordered set of crack pixels detected through the clustering and linking process. 

### 3.4. Crack Measurements

We measured the width of a crack for each crack pixel. Each crack pixel has an orientation and the width is measured in the perpendicular direction of the orientation of the crack-pixel. We assumed that two step-edges can be observed in the perpendicular direction. If those edges are aligned with the pixel boundaries, we are able to detect the edges by observing two neighboring pixels, but in most cases, the edges were not aligned to the pixel boundaries. Therefore, we observed more consecutive pixels along the perpendicular direction to the orientation of the crack segment and detected the edges. We used the following compositing equation, which explains the color composition at the boundary between the foreground and background [19]. In our case, the foreground color is the intensity of the crack (crack pixel), the background color is the intensity of non-crack pixels.

(11) C=αF+(1−α)B

where, 0≤α≤1.0.

**Figure 10:**
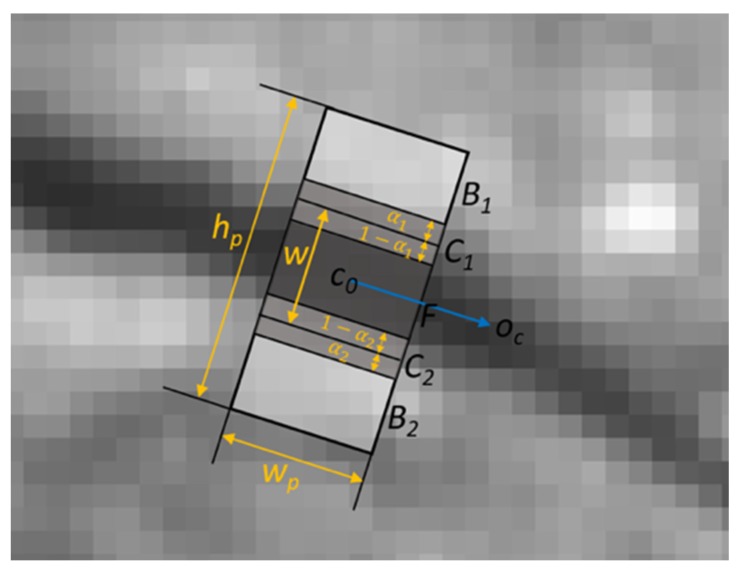
Crack width measurement.

The blending factor *α* can be computed by observing *F*, *B* and *C*, and this factor can provide us the information of whether how much portion of the composite pixel is from a crack or non-crack area (the physical area where the image is captured), as illustrated in Figure 10. *O_c_* is the orientation of a crack pixel, *C_0_*. In the figure, a rectangular patch centered at *C_0_* is rotated and aligned to *O_c_*. The location of composite pixels can be found by comparing *I*(*p*), the value of pixel *p*, and *I*(*p* + 2), the value of the second neighboring pixel of a pixel p. In the coordinates of the patch (left-bottom corner is the origin), if *I*(*p*) − *I*(*p* + 2) is the positive maximum and *I*(*p*) > *I*(*p* + 1) > *I*(*p* + 2), then the pixel, (*p* + 1) corresponds to the area *C_2_* in the Figure. If *I*(*p*) − *I*(*p* + 2) is the largest negative value and *I*(*p*) < *I*(*p* + 1) < *I*(*p* + 2), then the pixel, (*p* + 1) is *C_1_*. The blending factor *α*_1_ and *α*_2_ can be calculated by the image compositing Equation (11). The width of the crack pixel, *w* is the sum of the width of the area *F* and (1 − *α*_1_ + 1 − *α*_2_), as illustrated in Figure 10.

## 4. Experimental Results 

### 4.1. Data Collection

**Figure 11:**
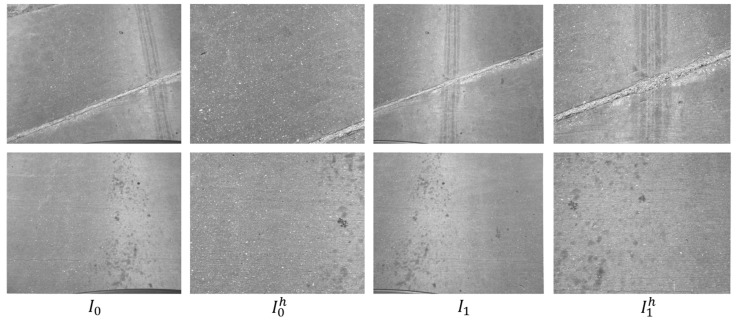
Images collected through our multi-view camera system. Each row represents one frame with four images and consists of two wide field-of-view images, *I*_0_ and *I*_1_, and two high-resolution images *I*_0_^h^, and *I*_1_^h^.

We selected a bridge in Nebraska for testing the proposed localization and crack mapping approach. We obtained 8 sequences along the longitudinal direction of the bridge. Each sequence consists of about 140 frames and each frame has four images from the four cameras. The resolution of each image is 2448 by 2048 pixels. The acquisition speed was approximately 20 miles per hour and the frame rate was set to 25 frames per second. Figure 11 shows some examples of the collected images.

### 4.2. Data Analytics and Visualization

**Figure 12:**
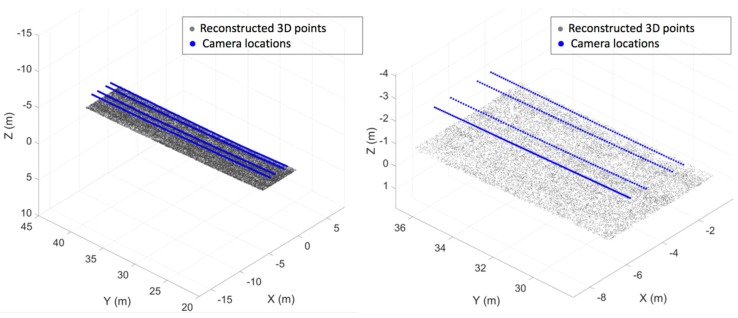
The reconstructed camera pose of all camera sequences and reconstructed 3D points (left image: zoom-out, right image: zoom-in).

**Figure 13:**
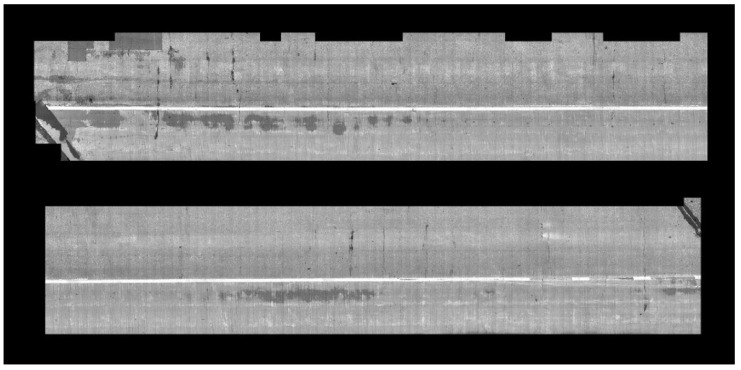
Global map with stitched images of the top surface of the entire bridge deck.

We implemented the localization and crack detection tasks for the data collected from the bridge. For the localization and 3D mapping, we implemented our own system based on ORB-SLAM, which takes a single-view sequence of images and performs simultaneous localization and mapping. Our system uses the ORB features and Bag-of-Words representation [15], and selects key-frames (which are a subset of a sequence) for localization and mapping. It detects loops and corrects any accumulated errors along the loops. Our implementation takes multiple sequences of multi-view frames. We calibrated the four cameras in which two of them have wide-angle views with overlapping images. We implemented stereo reconstruction and utilized the results for mapping and optimizing the estimated pose described in Section 3 of this paper. For each view, we extracted ORB features and generated its corresponding Bag-of-Words vector using DBoW2 [15]. With this image-level descriptor, we generated a graph by applying the proposed sequence matching strategy. The graph is traversed in breadth first order, but we employed a priority queue to visit the closest frame from the center of the bridge first, among all immediate neighbors of the visited frames. Figure 12 shows the reconstructed camera poses and 3D points, and Figure 13 shows the global map of the high-resolution images stitched together. The size of one pixel in the stitched map is 10 mils (0.25 mm).

**Figure 14:**
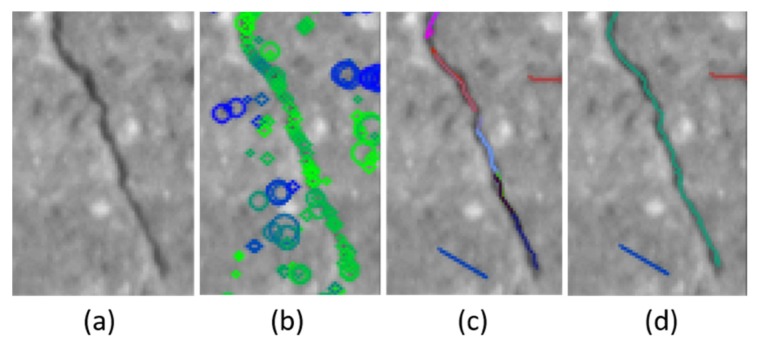
Detected crack pixels and generated crack segments: (**a**) input image, (**b**) detected crack pixels: blue colored circles represent crack pixels in a different direction than the green colored circles of crack pixels in one dominating orientation. The size of circle represents scale of the crack pixel, (**c**) represents initialized and extended crack segments, and (**d**) represents linked crack segments.

**Figure 15:**
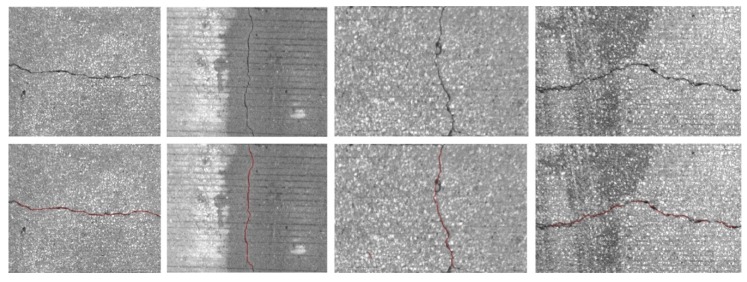
Cracks detected on the top surface of a bridge deck.

**Figure 16:**
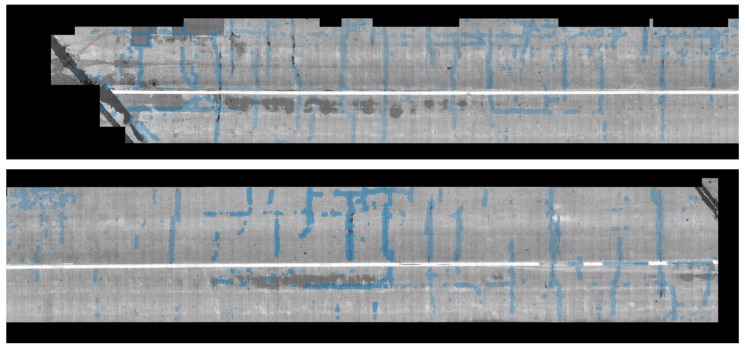
Global map of detected cracks on the top surface of the entire bridge deck.

After stitching all images to construct a global map, we implemented the crack detection method introduced in the methodology section. To detect the crack pixels, we did not examine all pixels but we checked one pixel that has a minimum intensity in each patch generated by a regular grid. A 5 by 5 patch was used in this experiment. Figure 14 and Figure 15 show examples of the detected cracks. Figure 16 shows the global map of detected transverse cracks. As mentioned earlier, these transverse cracks, caused by restrained shrinkage, are the main path where chlorides (from deicing salts) penetrate through and corrode the top and bottom of the steel reinforcement. This will eventually be the reason why bridge decks deteriorate.

### 4.3. Crack Database

We created a repository of JSON (JavaScript Object Notation) schemas (common ontologies) for deficiencies that bridge inspectors would be interested in. The schema repository is accessible at https://github.com/BridgingBigData/bridgehealthschema [10]. A crack database can be created by using the crack schema that identifies the location of the crack, average width of the crack, maximum width of the crack, number of cracks, spacing of cracks, and the length of the cracks. 

### 4.4. Results and Discussion

In order to compare our algorithms with similar methods (excluding the deep learning methods that were only implemented to a local region), we compared the detection performance between our proposed method and the STRUM classifier [1]. The reason we chose the STRUM classifier as the method for comparison is because this is the only research that actually implemented their method for the entire bridge deck, compared to other studies which only focused on local regions. The STRUM classifier uses the RANSAC (Random Sample Consensus) technique to fit a line segment on dark pixels of an image patch. Our proposed approach detects crack pixels and builds up crack segments. The crack pixel and crack segment representation are more flexible than a straight line because it can represent orientation changes and scale variances within a crack segment. 

We have collected 2638 image patches from crack and non-crack areas of the bridge. The dataset consists of 1262 crack image patches and 1376 background (non-crack) image patches. The STRUM classifier takes small image patches as input and labels them. Our approach does not necessarily take small image patches but we applied our approach to the same image patches for the comparison purpose. The Support Vector Machine (SVM) classifier with radial basis function kernel has been trained with the training data for both approaches. The parameters of SVM have been chosen empirically. With the 10-fold cross validation, the performance comparison showed 87.2% classification accuracy for the STRUM classifier, while 95.5% accuracy for the crack pixel-based representation introduced in our study. The results show that the crack pixel and crack segment-based representation can describe and identify more cracks than the STRUM classifier provides. 

**Figure 17:**
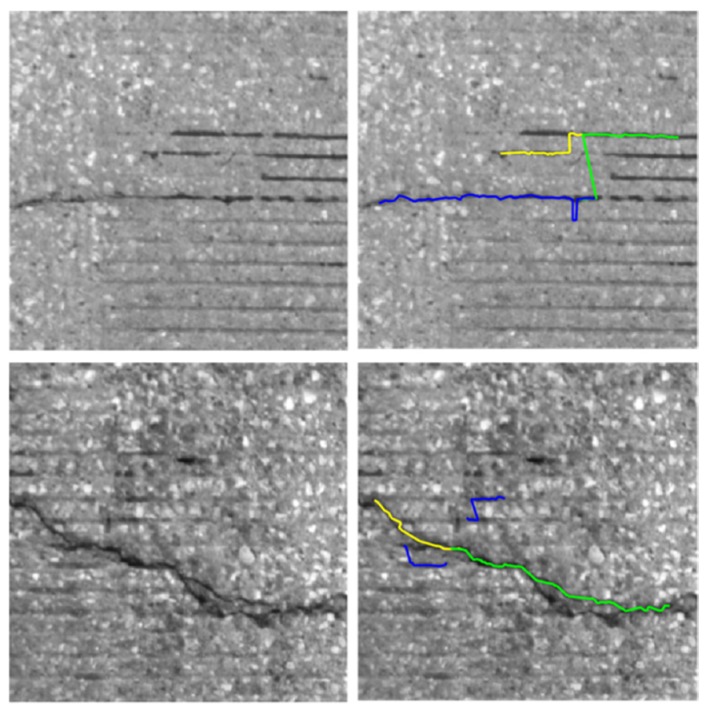
Examples of cases that did not indicate the crack properly.

During our training and implementation, crack pixels and segments were merged (green and blue lines shown on the top right image of Figure 17) with the wrong nearby crack pixels and segments if they were too close to each other and had consistent orientations. The left image is the input and the right image is the output of crack detection. This was found when we tested our methods on bridge decks that had tining marks. This error can be prevented by employing adaptive thresholds for each crack segment. 

In addition, if there are two close cracks (divided crack on the bottom left image), one of them is suppressed as shown in the bottom right image of the Figure 17 into one crack (green line). Relatively short crack segments (blue lines) in the bottom right image can be removed by post processing with relevant thresholds and only saving the segments with certain length, which becomes important in detecting transverse primary cracks. 

## 5. Conclusions

In this paper, we presented a new type of automated crack mapping system with details of each component of the Big Data image pipeline. We demonstrated that the system can localize multiple multi-view image sequences, detect cracks, and extract useful information from a real bridge site. The proposed automated transverse crack mapping system may serve as a useful inspection tool for big data analytics because the system is easy to deploy (no GPS or odometer data used) and provides multiple properties of cracks (average crack width, maximum crack width, number of primary transverse cracks, crack spacing, and location of crack) in both spatial and time scale. Any progression of the crack deterioration can be checked and compared if the system is deployed multiple times. Our proposed methodology was compared with a different method that was implemented to an entire bridge deck, which is the goal of our research. Our crack pixel and crack segment-based representation for the detection demonstrated that it can identify cracks with 95.5% accuracy for the 2638 image patches compared. 

For future work, we will extend this system to inspect the bottom of the bridge deck to identify the full-depth cracks that penetrate through the bridge deck. Since our system estimates 3D poses and 3D points, the extension will be straightforward. The acquisition will be completed by cameras attached to a flying object. The identical localization and mapping techniques introduced in this study will be adapted for the automated flight control of the system because GPS signals will not be available under a bridge deck.
